# The novel adipocytokine visfatin exerts direct cardioprotective effects

**DOI:** 10.1111/j.1582-4934.2008.00332.x

**Published:** 2008-04-08

**Authors:** Shiang Y Lim, Sean M Davidson, Ajeev J Paramanathan, Christopher C T Smith, Derek M Yellon, Derek J Hausenloy

**Affiliations:** The Hatter Cardiovascular Institute, University College London Hospital and Medical SchoolLondon, United Kingdom

**Keywords:** visfatin, ischaemia, reperfusion, cardioprotection

## Abstract

Visfatin is an adipocytokine capable of mimicking the glucose-lowering effects of insulin and activating the pro-survival kinases phosphatidylinositol-3-OH kinase (PI3K)-protein kinase B (Akt) and mitogen-activated protein kinase kinase 1 and 2 (MEK1/2)-extracellular signal-regulated kinase 1 and 2 (Erk 1/2). Experimental studies have demonstrated that the activation of these kinases confers cardioprotection through the inhibition of the mitochondrial permeability transition pore (mPTP). Whether visfatin is capable of exerting direct cardioprotective effects through these mechanisms is unknown and is the subject of the current study. Anaesthetized C57BL/6 male mice were subjected to *in situ* 30 min. of regional myocardial ischaemia and 120 min. of reperfusion. The administration of an intravenous bolus of visfatin (5 × 10^−6^μmol) at the time of myocardial reperfusion reduced the myocardial infarct size from 46.1 ± 4.1% in control hearts to 27.3 ± 4.0% (*n*≥ 6/group, *P* < 0.05), an effect that was blocked by the PI3K inhibitor, wortmannin, and the MEK1/2 inhibitor, U0126 (48.8 ± 5.5% and 45.9 ± 8.4%, respectively, *versus* 27.3 ± 4.0% with visfatin; *n*≥ 6/group, *P* < 0.05). In murine ventricular cardiomyocytes subjected to 30 min. of hypoxia followed by 30 min. of reoxygenation, visfatin (100 ng/ml), administered at the time of reoxygenation, reduced the cell death from 65.2 ± 4.6% in control to 49.2 ± 3.7%*(n* > 200 cells/group, *P* < 0.05), an effect that was abrogated by wortmannin and U0126 (68.1 ± 5.2% and 59.7 ± 6.2%, respectively; *n* > 200 cells/group, *P* > 0.05). Finally, the treatment of murine ventricular cardiomyocytes with visfatin (100 ng/ml) delayed the opening of the mPTP induced by oxidative stress from 81.2 ± 4 sec. in control to 120 ± 7 sec. (*n* > 20 cells/group, *P* < 0.05) in a PI3K- and MEK1/2-dependent manner. We report that the adipocytokine, visfatin, is capable of reducing myocardial injury when administered at the time of myocardial reperfusion in both the *in situ* murine heart and the isolated murine cardiomyocytes. The mechanism appears to involve the PI3K and MEK1/2 pathways and the mPTP.

## Introduction

Visceral fat accumulation, a key feature of the metabolic syndrome, is associated with the development of diabetes mellitus, a three-fold increased risk of developing coronary heart disease [1], a two to three times increase in cardiovascular mortality [2] and worse clinical outcome following an acute myocardial infarction [3–5] or a primary percutaneous coronary intervention [6]. Although formerly regarded as purely an energy storage site, the emerging studies suggest that adipose tissue is an active endocrine organ producing ‘adipocytokines’, hormones that influence a diverse array of processes including appetite and energy balance, immunity, insulin sensitivity, angiogenesis, blood pressure, lipid metabolism and haemostasis, all factors that can impact cardiovascular disease [7]. In this regard, the adipocytokines, adiponectin [8], leptin [9] and apelin [10], have been linked to cardioprotection in recent experimental studies.

The recently discovered adipocytokine, visfatin, has been demonstrated to mimic the glucose-lowering effect of insulin and improve insulin sensitivity [11]. Furthermore, by binding to the insulin receptor, visfatin has been demonstrated to activate intra-cellular kinase signalling cascades, such as the PI3K-Akt and mito-gen-activated protein kinase (MAPK) pathways [11], through which it may exert an antiapoptotic effect [12]. Experimental studies have indicated that the activation of pro-survival protein kinas-es such as PI3K-Akt and MEK1/2-Erk1/2 MAPK, at the time of myocardial reperfusion [13, 14], confers powerful cardioprotec-tion, an effect attributable, in part, to the inhibition of the mito-chondrial permeability transition pore (mPTP) [15]. The mPTP is a non-specific mitochondrial channel whose opening in the first few minutes of myocardial reperfusion is a critical determinant of the cardiomyocyte death [16, 17]. In addition, several studies have reported that the visfatin gene is induced in response to hypoxia, an effect mediated by hypoxia-inducible factor [18, 19], raising the possibility that visfatin is up-regulated in response to myocardial ischaemia. In the current study, we have suggested that visfatin elicits cardioprotection through the activation of PI3K-Akt and MEK1/2-Erk1/2 and the subsequent inhibition of mPTP opening.

## Methods

### Animals and materials

Experiments were carried out in accordance with the United Kingdom Home Office Guide on the Operation of Animal (Scientific Procedures) Act of 1986. C57BL/6 male mice were obtained from Charles River UK Limited (Margate, UK). Wortmannin (Tocris Bioscience, Bristol, UK), U0126 (Tocris Bioscience) and cyclosporin A (CsA) (Calbiochem, San Diego, CA, USA) were dissolved in dimethyl sulfoxide (DMSO) (0.02%). Visfatin (Alexis Biochemical, Lausen, Switzerland) was dissolved in normal saline.

### *In vivo* murine model of acute myocardial infarction

C57BL/6 male mice (8–12 weeks of age and weighing 25–30 g) were anaesthetized by intraperitoneal injection with a combination of ketamine, xylazine and atropine (0.01 ml/g; the final concentrations of ketamine, xylazine and atropine were 10 mg/ml, 2 mg/ml and 0.06 mg/ml, respectively) and their body temperature was maintained at 37°C. The external jugular vein and carotid artery were isolated and cannulated for drug administration and mean arterial blood pressure (MABP) measurement, respectively. A tracheotomy was performed for artificial respiration at 120 strokes/min. and 200 μl stroke volume using a rodent Minivent (type 845; Harvard Apparatus, Kent, United Kingdom) and supplemental oxygen was supplied. A limb lead I electrocardiogram (ECG) was recorded. A left anterior thoracotomy and a chest retractor were used to expose the heart. Ligation of the left anterior descending (LAD) coronary artery was performed ∼2 mm below the tip of the left atrium using an 8/0 prolene monofilament polypropylene suture. Successful LAD coronary artery occlusion was confirmed by the presence of ST elevation and a decrease in the arterial blood pressure. At the end of the reperfusion, the heart was isolated and the aortic root was cannulated and used to inject 2,3,5-triph-enyltetrazolium chloride (TTC, 5 ml of 1%) in order to demarcate the infarcted tissue. The LAD coronary artery was then re-ligated and Evans blue dye (2 ml of 0.5%) was perfused to delineate the area at risk (AAR). The heart was frozen and sectioned perpendicular to the long axis (1- to 2-mm thick). The slices were then transferred to 10% neutral buffer formalin for 2 hrs at room temperature to stabilize the staining. The AAR and infarct size were determined by computerized planimetry performed with the National Institutes of Health (NIH) software Image (Bethesda, MD, USA). The AAR was expressed as a percentage of the left ventricle and the infarct size was expressed as a percentage of the AAR [20].

### Experimental protocol for murine myocardial infarction studies

**Fig. 1:**
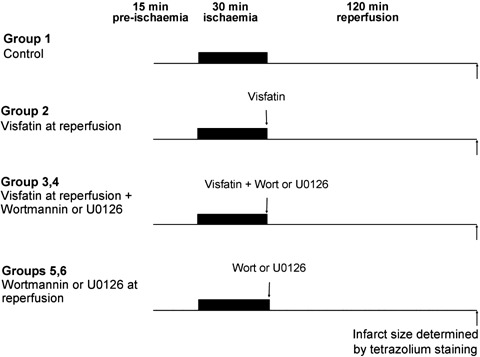
Experimental protocols for *in vivo* murine infarction studies. All hearts were subjected to 15 min. of stabilization, 30 min. of regional myocardial ischaemia, followed by 120 min. of myocardial reperfusion at the end of which the infarct size was determined by tetrazolium staining. Visfatin was given immediately prior to the onset of myocardial reperfusion in the presence or absence of the PI3K and MEK1/2 inhibitors wortmannin and U0126, respectively.

C57BL/6 male mice were randomly assigned to one of the following six treatment groups (Fig. 1). The hearts were subjected to 30 min. of ischaemia, followed by 120 min. of reperfusion at the end of which the infarct size was determined by tetrazolium staining.

1) Vehicle control (*n*= 13): either 0.02% DMSO or normal saline (0.1 ml) was administered immediately prior to the onset of myocardial reperfusion.
2) Visfatin (*n*= 8): an intravenous bolus of Visfatin (5 × 10^−6^μmol, 0.1 ml) dissolved in normal saline was administered immediately prior to the onset of myocardial reperfusion.
3) Visfatin + wortmannin (*n*= 6): visfatin was administered in the presence of wortmannin (15 μg/kg) dissolved in DMSO (0.02%) immediately prior to the onset of reperfusion.
4) Vistatin + U0126 (*n*= 6): visfatin was administered in the presence of U0126 (0.2 mg/kg) dissolved in DMSO (0.02%) immediately prior to the onset of reperfusion.
5,6) Wortmannin or U0126 (*n*= 4): wortmannin or U0126 was administered alone immediately prior to the onset of reperfusion.

### Experimental protocol for murine ventricular cardiomyocyte isolation

The mice were injected with heparin sodium (250 IU) and anaesthetized with ketamine/xylazine/atropine. The hearts were rapidly excised, cannulated and perfused with (in mmol/L) 113.0 NaCl, 4.7 KCl, 0.6 KH_2_PO_4_, 0.6 Na_2_HPO_4_, 1.2 MgSO_4_.7H_2_O, 12.0 NaHCO_3_, 10.0 KHCO_3_, 30.0 taurine, 10.0 HEPES, and 5.5 glucose, saturated with 95% O_2_–5% CO_2_ at 37°C. The hearts were perfused at 3 ml/min. with perfusion buffer for 4 min., then with perfusion buffer containing 0.9 mg/ml collagenase type II (Worthington Biochemical Corporation, Lakewood, NJ, USA) 0.125 mg/ml hyaluronidase and 12.5 ixmol/L CaCl_2_ for 10 min. The ventricles were then cut into several pieces and shaken at 37°C, with oxygenation for 10 min. The supernatant was collected and 5% foetal calf serum was added. After centrifugation at 600 rpm for 3 min., the cell pellet was suspended in 10 ml of perfusion buffer containing 12.5 μmol/L CaCl_2_ and the calcium concentration was gradually restored to 1 mmol/L over 20 min. The myocytes were re-centrifuged, then seeded onto sterilized laminin-coated coverslips in minimum essential medium (MEM) containing 10 units/ml penicillin, 10 ixg/ml streptomycin and 5% foetal calf serum for incubation before use on the same day of isolation.

### Experimental protocol for ventricular cardiomyocyte viability studies

All cells were subjected to 30 min. of hypoxia, then 30 min. of reoxygena-tion to simulate ischaemia-reperfusion injury. Hypoxia was induced in a custom-made airtight hypoxic chamber using a buffer simulating the conditions of ischaemia (in mmol/L: 1.0 KH_2_PO4, 10.0 NaHCO_3_, 1.2 MgCl_2_.6H_2_0, 25.0 Na(4-(2-hydroxyethyl)-1-piperazineethanesulfonic acid) (HEPES), 74.0 NaCl, 16.0 KCl, 1.2 CaCl_2_ and 20.0 Na lactate, pH 6.7) bubbled with 95% nitrogen/5% CO_2_. Reoxygenation was achieved by replacing the buffer with a solution containing (in mmol/L) 1.0 KH_2_PO4, 10.0 NaHCO_3_, 1.2 MgCl_2_.6H_2_0, 25.0 NaHEPES, 98.0 NaCl, 3.0 KCl, 1.2 CaCl_2_ 10.0 D-glucose, 2.0 Na pyruvate, pH 7.4, bubbled with 95% O2/5% CO_2_. The cells were randomized to the following six treatment groups (*n* > 200 cells per group from three to six different animals).

1) Vehicle control: cells were reoxygenated in normal buffer or buffer containing 0.02% DMSO.
2) Visfatin: cells were reoxygenated in buffer containing visfatin (100 ng/ml).
3) Visfatin + wortmannin: cells were reoxygenated in buffer containing visfatin and wortmannin (100 nmol/L).
4) Vistatin + U0126: cells were reoxygenated in buffer containing visfatin and U0126 (10 μmol/L).
5,6) Wortmannin or U0126: Cells were reoxygenated in buffer containing either wortmannin or U0126.

At the end of the reoxygenation, 5 μl of propidium iodide (PI, 1 μg/ml) was added to the cells for 5 min. followed by 1 ml of 10% neutral buffer formalin. The percentage of dead cells (as indicated by red fluorescence, PI-positive) in duplicate wells was calculated by fluoroscence microscopy and was expressed as a percentage of the total number of cardiomyocytes (PI-positive and PI-negative).

### Assay of mPTP opening

The sensitivity of the mPTP to opening was assayed using a well-characterized and reproducible cellular model [9, 15]. Live isolated myocytes were incubated with the fluorescent dye tetra-methyl rhodamine methyl ester (TMRM, 3 μmol/L) for 15 min. in microscopy buffer *(i.e.* perfusion buffer containing 1.2 mmol/L CaCl_2_), then washed and imaged using con-focal microscopy. TMRM, a lipophilic cation, accumulates selectively into the mitochondria according to the mitochondrial membrane potential. Continual confocal laser scanning generates reactive oxygen species (ROS) from the TMRM within the mitochondria that, after several minutes, provoke mPTP opening, as indicated by the mitochondrial depolarization. After loading with TMRM, and before confocal laser illumination, the cardiomyocytes were randomly assigned to the following treatment groups (*n* > 20 cells per group from three to five different mice):

1) Vehicle control: cardiomyocytes were bathed in buffer containing either 0.02% DMSO or normal saline.
2) Visfatin: cardiomyocytes were treated with visfatin (100 ng/ml) for 5 min.
3) Visfatin + wortmannin: cardiomyocytes were treated with visfatin and wortmannin (100 nmol/L) for 5 min.
4) Vistatin + U0126: cardiomyocytes were treated with visfatin and U0126 (10 μmol/L) for 5 min.
5,6) Wortmannin or U0126: cardiomyocytes were treated with either wortmannin or U0126 for 5 min.
7) CsA: cardiomyocytes were treated with CsA (0.4 μmol/L) for 5 min.

### Statistical analysis

All values are expressed as mean ± standard error of mean (S.E.M.). Infarct size to AAR, cell viability data and the time taken to induce mPTP opening were analysed by one-way anova followed by Dunnett's multiple comparison *post-hoc* test. The differences were considered significant when *P* < 0.05.

## Results

In general, except for the increase in MABP in the wortmannin-treated hearts, the treatment with visfatin and/or the kinase inhibitors had no significant effects on the haemodynamic parameters of MABP and heart rate (Table 1).

**Table 1:** Haemodynamic variables in mice treated with vehicle, visfatin ± wortmannin or U0126 at reperfusion

Mean Arterial Blood Pressure (mmHg)					

	Time-Point				

	I, 0 min.	I, 15 min.	R, 5 min.	R, 30 min.	R, 120 min.
**Control**	119 ± 4	98 ± 4	98 ± 4	85 ± 3	49 ± 4
**Visfatin**	114 ± 6	90 ± 6	84 ± 8	80 ± 4	51 ± 5
**Visfatin + wort**	110 ± 4	91 ± 3	103 ± 5	83 ± 6	56 ± 7
**Visfatin + U0**	120 ± 4	102 ± 4	99 ± 5	85 ± 2	64 ± 3
**Wort**	123 ± 7	102 ± 7	122 ± 9*	101 ± 4*	47 ± 8
**U0**	115 ± 4	101 ± 4	101 ± 2	84 ± 4	53 ± 8

Heart Rate (bpm)					

	Time-Point				

	I, 0 min.	I, 15 min.	R, 5 min.	R, 30 min.	R, 120 min.
**Control**	354 ± 9	349 ± 10	344 ± 7	354 ± 9	388 ± 15
**Visfatin**	369 ± 9	334 ± 10	337 ± 11	340 ± 6	370 ± 9
**Visfatin + wort**	343 ± 11	329 ± 5	332 ± 12	337 ± 10	362 ± 14
**Visfatin + U0**	351 ± 12	341 ± 15	347 ± 16	366 ± 14	395 ± 8
**Wort**	385 ± 19	361 ± 12	384 ± 17	373 ± 14	386 ± 14
**U0**	354 ± 16	340 ± 9	362 ± 12	378 ± 18	428 ± 25

Mean arterial blood pressure (MABP) and heart rate (HR) were taken at 0 and 15 min. into occlusion (I, 0 min. and I, 15 min.), and at 5, 30 and 120 min. into reperfusion (R, 5 min.; R, 30 min. and R, 120 min.). Wort, Wortmannin; U0, U0126; bpm, beats per minute. * indicates *P***<** 0.05 *versus* control.

### Visfatin reduces the myocardial infarct size *in vivo* when administered at the time of reperfusion through PI3K and MEK1/2

**Fig. 2:**
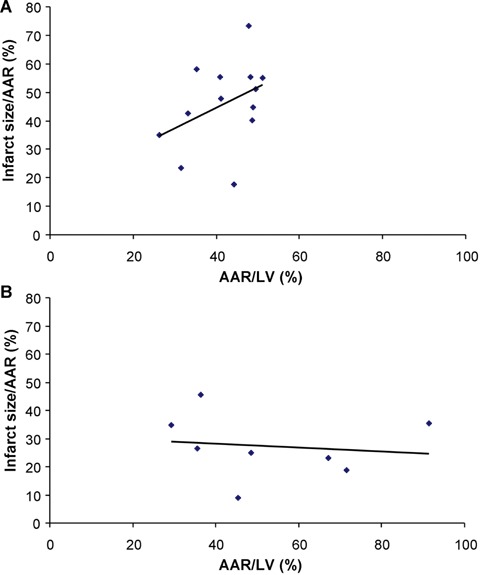
Infarct size, expressed as a percentage of the area at risk (AAR), in hearts administered visfatin (5 × 10^−6^μmol) at the time of myocardial reperfusion, in the presence or absence of the PI3K and MEK1/2 inhibitors wortmannin (Wort, 15 μg/kg) and U0126 (0.2 mg/kg), respectively. Visfatin is shown to reduce the myocardial infarct size significantly, and this cardioprotective effect is abolished in the presence of the kinase inhibitors. Numbers in parentheses indicate *n* numbers. *P < 0.05.

**Fig. 3:**
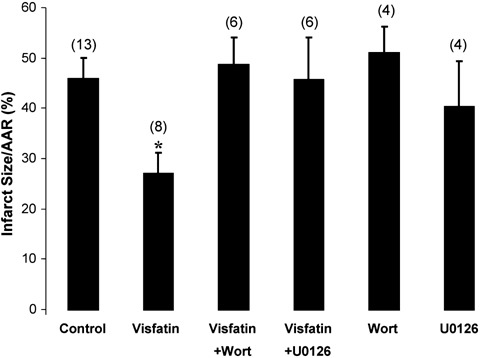
Infarct size plotted against the area at risk (AAR) for control hearts (**A**) and visfatin-treated hearts (**B**).

The AAR of myocardial infarction was comparable among the treatment groups (42.1 ± 2.0% in control *versus* 53.2 ± 8.0% visfatin, 53.6 ± 8.0% visfatin + wortmannin, 47.2 ± 5.0% visfatin + U0126, 48.2 ± 3.0% wortmannin, 49.2 ± 9.0% U0126; *P* > 0.05). Visfatin, given at the time of myocardial reperfusion, reduced the infarct size from 46.1 ± 4.1% in control to 27.3 ± 4.0% (*P* < 0.05; Fig. 2). Wortmannin and U0126 both abolished the decrease in the infarct size observed in the visfatin-treated hearts (46.1 ± 4.1% in control *versus* 48.8 ± 5.5% with visfatin + wortmannin and 45.9 ± 8.4% with visfatin and U0126; Fig. 2). The administration of wortmannin or U0126 alone had no effect on the infarct size (46.1 ± 4.1% in control *versus* 51.2 ± 5.1% with wortmannin and 40.5 ± 8.9% with U0126; *P* > 0.05; Fig. 2). The infarct size is plotted against the AAR in Fig. 3.

### Visfatin reduces the cardiomyocyte death when administered at the time of reoxygenation through PI3K and MEK1/2

**Fig. 4:**
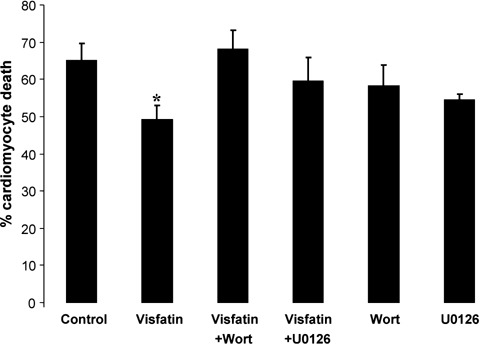
Cell viability following 30 min. of hypoxia and 30 min. of reoxygenation in murine ventricular cardiomyocytes treated with visfatin (100 ng/ml) in the presence or absence of the PI3K and MEK1/2 inhibitors wortmannin (Wort, 100 nmol/L) and U0126 (10 μmol/L), respectively. Visfatin reduces the percentage cardiomyocyte death elicited by hypoxia-reoxygenation injury, and this cardioprotective effect is abolished in the presence of the kinase inhibitors. *n* > 200 cells per group. *P < 0.05.

In murine ventricular cardiomyocytes subjected to an episode of sustained hypoxia, the presence of visfatin at the time of reoxygenation reduced the cell death from 65.2 ± 4.6% in control to 49.2 ± 3.7% with visfatin (*P* < 0.05; Fig. 4). The cardioprotec-tive effect of visfatin was abrogated in the presence of wortmannin and U0126 (65.2 ± 4.6% in control *versus* 68.1 ± 5.2% with visfatin **+** wortmannin and 59.7 ± 6.2% with visfatin + U0126; *P* > 0.05; Fig. 4). The kinase inhibitors alone did not influence the cardiomyocyte viability (65.2 ± 4.6% in control *versus* 58.3 ± 5.5% with wortmannin and 54.5 ± 1.4% with U0126; *P* > 0.05; Fig. 4).

### Visfatin delays the mPTP opening in a PI3K- and MEK1/2-dependent manner

**Fig. 5:**
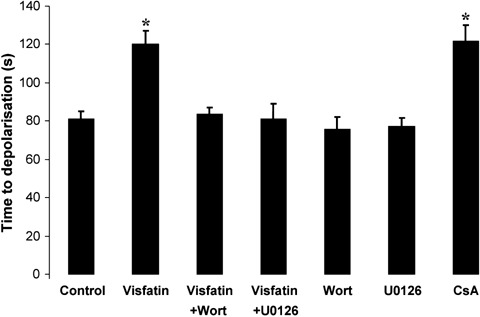
The time taken to induce mitochondrial permeability transition pore (mPTP) opening in murine ventricular cardiomyocytes treated with visfatin (100 ng/ml) in the presence or absence of the PI3K and MEK1/2 inhibitors wortman-nin (Wort, 100 nmol/L) and U0126 (10 μmol/L), respectively. Visfatin delays the time taken to induce mPTP opening, an effect that is abolished in the presence of the kinase inhibitors. *n* > 20 cells per group. *P < 0.01.

The treatment of cardiomyocytes with visfatin delayed the time taken to induce mPTP opening from 81.2 ± 4.0 sec. in control to 120.0 ± 6.5 sec. with visfatin (*P* < 0.01; Fig. 5). The effect of visfatin on the mPTP opening was comparable in magnitude to treatment with CsA (the archetypal mPTP inhibitor and a positive control) (81.2 ± 4.0 sec. in control *versus* 121.5 ± 8.4 sec. with CsA; *P* < 0.01; Fig. 5). The inhibitory effect of visfatin on the mPTP opening was abrogated in the presence of the PI3K and MEK1/2 inhibitors, wortmannin and U0126, respectively (83.4 ± 3.4 sec. with visfatin + wortmannin and 80.9 ± 8.1 sec. with visfatin + U0126; *P* > 0.05; Fig. 5). The kinase inhibitors alone did not influence the cardiomyocyte mPTP opening (81.2 ± 4.0 sec. in control *versus* 75.7 ± 6.5 sec. with wortmannin and 77.0 ± 4.8 sec. with U0126; *P* > 0.05; Fig. 5).

## Discussion

The pivotal findings of the current study are as follows: (1) we demonstrate for the first time that the administration of the novel adipocytokine, visfatin, specifically at the time of myocardial reperfusion, dramatically reduces the myocardial infarct size using an *in vivo* murine infarction model; (2) the cardioprotective effect induced by visfatin is demonstrated to be a direct cellular effect, as demonstrated by the improved cardiomyocyte viability observed using isolated cardiomyocytes treated with visfatin at the time of reoxygenation; (3) visfatin treatment delays the opening of the mPTP in isolated cardiomyocytes subjected to oxidative stress and (4) importantly, the cardioprotective effect is dependent on the activation of the pro-survival kinases, PI3K and MEK1/2.

Visfatin has only recently been discovered as an adipocy-tokine, and as such, its pathophysiological role, in common with some of the other newer adipocytokines, remains to be determined. Fukuhara *et al.*[11] first identified visfatin in 2005, as an adipocytokine that appeared to lower the serum glucose by mimicking the actions of insulin, thereby raising the exciting possibility of a novel antidiabetic agent [11]. In the same study, the authors demonstrated that visfatin phosphorylated the insulin receptor, insulin receptor substrate-1 (IRS-1) and -2 (IRS-2), PI3K, Akt and MAPK [11]. However, whether visfatin actually mediates its effects by binding to the insulin receptor has been recently debated [21].

Given that visfatin has the ability to activate Akt and MAPK, and the prior knowledge that these kinases belong to the reperfusion injury salvage kinase (RISK) pathway [13, 14], a group of protein kinases that on activation at the time of myocardial reperfusion confer powerful cardioprotection, we were keen to determine whether visfatin was able to confer protection against acute myocardial ischaemia-reperfusion injury. In the present study, although we demonstrated that specific pharmacological blockers of the RISK pathway abolished the protection elicited by visfatin, a limitation of our study is that we did not determine whether visfatin treatment resulted in the phosphorylation of kinases of Akt and Erk1/2. Given that the cardioprotection mediated through the RISK pathway has been linked to the inhibition of the mPTP [15], a non-specific high-conductance channel of the inner mitochondr-ial membrane that is believed to mediate lethal reperfusion injury by opening in the first few minutes of myocardial reperfusion [16], it was important in the current study to link the protective effect of visfatin with the inhibition of mPTP opening. It must be appreciated that although the model we used to detect mPTP opening in the current study is a well-established and reproducible model [9, 15], it was limited by the fact that it only reproduced the oxidative stress component of ischaemia-reperfusion injury to provoke mPTP opening.

As described above, the pathophysiological role of visfatin is currently unclear. The clinical studies in which plasma levels of visfatin were measured in different patient groups showed no clear correlations. Indeed, measurements of plasma visfatin levels in human beings have yielded conflicting results, with raised levels being reported in obese children [22], diabetic patients [23, 24], patients after weight loss mediated by gastroplastic surgery [25] and patients being treated with thiazolidinedione and rosiglitazone [26], whilst other studies have reported reduced levels with gestational diabetes [27], exercise in patients with type 1 diabetes [28], massive weight loss [29] and obesity [30].

Interestingly, visfatin was formerly identified as a pre-B cell colony-enhancing factor (PBEF) [31], a growth factor for early B cells, which has been linked to a diverse variety of cellular processes, with studies demonstrating PBEF to (a) act as a biomarker of acute lung injury [32, 33], (b) be up-regulated in infected foetal membranes [34], (c) inhibit neutrophil apoptosis in experimental inflammation and clinical sepsis [12] and (d) participate in the maturation of vascular smooth muscle cells through a nicotinamide adenine dinucleotide (NAD^+^)-dependent mechanism [35].

Intriguingly, PBEF/visfatin has also been identified as the enzyme nicotinamide phosphoribosyl transferase (Nampt), the rate-limiting enzyme in NAD biosynthesis that mediates the conversion of nicotinamide to nicotinamide mononucleotide [36]. Given the pivotal role of Nampt in NAD biosynthesis, pharmacological inhibitors of Nampt such as FK866 are currently being investigated as potential novel anticancer agents [37, 38]. Clearly, further studies are required to elucidate the pathophysiological role of visfatin in relation to these previous identities of PBEF and Nampt. For example, the possibility that the cardioprotective effect elicited by visfatin could be due to the potentiation of Nampt, which would be expected to up-regulate NAD biosynthesis, thereby enhancing energy metabolism and redox biochemistry, factors that underpin the tolerance of myocardial tissue to ischaemic injury, requires investigation.

Whether endogenous visfatin contributes to cardioprotection in the clinical setting is unclear; but in this regard, it is interesting that the experimental studies have reported that the visfatin gene is induced in response to hypoxia, an effect mediated by hypoxia-inducible factor [18, 19], raising the possibility that visfatin may be up-regulated in response to myocardial ischaemia. Thus, further studies are required to ascertain the plasma levels of visfatin in patients presenting with an acute myocardial infarction. The recent investigations have linked visfatin with MAPK-mediated angiogenesis [39] as well as a potential pro-inflammatory mediator in unstable atherosclerotic plaques, suggesting that endogenous visfatin may have a detrimental effect in coronary artery disease [40]. In the current study, we found that the acute administration of exogenous visfatin can protect against acute myocardial ischaemia-reperfusion injury in a non-atherosclerotic animal model of myocardial infarction and in isolated cardiomyocytes. Further studies will be required to determine whether exogenous visfatin elicits the same cardioprotective benefits in the atherosclerotic models of myocardial infarction.

In summary, this study has demonstrated, for the first time, that the novel adipocytokine, visfatin, has the ability to reduce the myocardial infarct size when administered at the time of myocardial reperfusion. This powerful cardioprotective effect appears to be a direct cellular effect that is mediated through the activation of PI3K and MEK1/2 and the inhibition of mPTP opening.
